# A Genome-Wide Association Study Identifies Risk Loci to Equine Recurrent Uveitis in German Warmblood Horses

**DOI:** 10.1371/journal.pone.0071619

**Published:** 2013-08-14

**Authors:** Maike Kulbrock, Stefanie Lehner, Julia Metzger, Bernhard Ohnesorge, Ottmar Distl

**Affiliations:** 1 Institute for Animal Breeding and Genetics, University of Veterinary Medicine Hannover, Hannover, Germany; 2 Clinic for Horses, University of Veterinary Medicine Hannover, Hannover, Germany; Temasek Life Sciences Laboratory, Singapore

## Abstract

Equine recurrent uveitis (ERU) is a common eye disease affecting up to 3–15% of the horse population. A genome-wide association study (GWAS) using the Illumina equine SNP50 bead chip was performed to identify loci conferring risk to ERU. The sample included a total of 144 German warmblood horses. A GWAS showed a significant single nucleotide polymorphism (SNP) on horse chromosome (ECA) 20 at 49.3 Mb, with *IL-17A* and *IL-17F* being the closest genes. This locus explained a fraction of 23% of the phenotypic variance for ERU. A GWAS taking into account the severity of ERU, revealed a SNP on ECA18 nearby to the crystalline gene cluster *CRYGA*-*CRYGF*. For both genomic regions on ECA18 and 20, significantly associated haplotypes containing the genome-wide significant SNPs could be demonstrated. In conclusion, our results are indicative for a genetic component regulating the possible critical role of IL-17A and IL-17F in the pathogenesis of ERU. The associated SNP on ECA18 may be indicative for cataract formation in the course of ERU.

## Introduction

Autoimmune diseases are characterized by an aberrant immune response. More than 35 cytokine and cytokine receptor gene loci have emerged as risk factors for over 15 autoimmune diseases in human [1]. Particularly, genome-wide association studies (GWAS) have facilitated the identification of susceptibility factors for autoimmune diseases. Equine recurrent uveitis (ERU) has been linked with human autoimmune diseases [2]–[6]. This equine ocular disease may serve as a spontaneous model for human autoimmune uveitis [2], [5], [6]. ERU is a common eye disease and the most common cause of blindness in horses [2], [7]–[9]. Several studies indicated prevalences of ERU in Western Europe horse populations from 3 to 15% [7]–[10]. Horses affected by ERU show recurrent-remitting episodes of intraocular inflammations [2], [3], [7]–[12]. Acute clinical signs of ERU include blepharospasm, photophobia, lacrimation, corneal haze, pupil miosis, aqueous flare, hypopyon or hyphema [7]–[12]. In the long-term, further changes become visible in ERU-affected horses such as synechiae, pigment deposition on the anterior lens capsule, phthisis bulbi, retinal detachment, cataract and lens luxation [7]–[12]. These profound damages lead to blindness or amblyopia, causing premature retirement of horses and high economic losses for the owners. The diagnosis of ERU is based on the characteristic clinical signs with recurrent or persistent inflammations of one or both eyes [7]–[12]. Until present, the aetiology of ERU is not yet fully understood. In horses with ERU, the majority of direct and indirect vitreal sample analyses are positive to *Leptospira* spp. without presenting the horses any common clinical signs of systemic leptospirosis [13]–[19]. The link between *Leptospira* spp. and the chronic recurrence of intraocular inflammations have driven the hypothesis that ERU may be considered as an autoimmune uveitis [9], [10], [20].

The predominant presence of CD4+ T-cells, an increased transcription of interleukin 2 (IL–2) and interferon–γ (IFNG) and low IL–4 mRNA expression in ERU-affected eyes suggest that ERU is a Th1-like-lymphocyte-mediated autoimmune disease [21]–[24]. Furthermore, immunoreactivity for IL-6, IL-17, and IL-23, in conjunction with T lymphocytes as predominating inflammatory cells, suggests that IL-17-secreting helper T-cells play a role in pathogenesis of ERU [25].

In the United Kingdom, cases with ERU became rare 40 years after abandonment of breeding with stallions showing cataracts [26]. Appaloosas often have persistent uveitis and show rapid progression, thus being more susceptible to suffer unilateral or bilateral blindness secondary to uveitis. This susceptibility is 3.8 times greater than in non-Appaloosas with uveitis [2], [27]. In Germany, warmblood horses and ponies are more often affected by ERU than thoroughbreds [9]. Serological studies showed an association among ERU-affected horses and the MHC class I haplotype ELA-A9 suggesting hereditary components being involved in ERU [28]. In Appaloosa, each one allele of the two microsatellites located within the equine MHC region was found to be overrepresented in ERU-affected Appaloosas compared to unaffected Appaloosas [29]. Therefore, both class I and class II equine MHC loci may contribute to the susceptibility for ERU in Appaloosas [29]. A potential involvement of toll-like-receptors (TLR) in the pathogenesis of ERU was shown using expression analysis. TLR-2 and TLR-9 mRNA were significantly increased in the ciliary body and TLR-2 also in the iris of ERU-diseased eyes compared with normal eyes [30].

The objective of the present study was to perform a genome-wide association study for ERU in German warmblood horses using the Illumina equine SNP50 beadchip. In the genomic regions identified through the GWAS, we determined positional candidate genes by their possible role in ERU.

## Results

**Figure 1:**
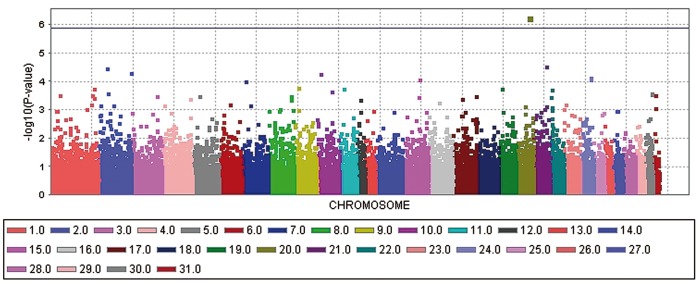
Manhattan plot of –log_10_P-values of the case-control genome-wide association study for equine recurrent uveitis (ERU) in German warmblood horses using a general linear model analysis. On the X-axis, the SNPs are given by horse chromosome number. The –log_10_P-values for each SNP effect are plotted against the SNP position on each chromosome. Chromosomes are differentiated by colors. The color keys are given below the plot. The blue line indicates the threshold of the –log_10_P-values for genome-wide significance after correcting for multiple testing.

**Figure 2:**
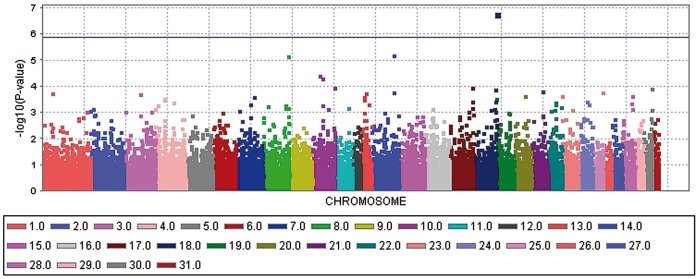
Manhattan plot of –log_10_P-values of the genome-wide association study for the severity score of equine recurrent uveitis (ERU) in German warmblood horses using a general linear model analysis. On the X-axis, the SNPs are given by horse chromosome number. The –log_10_P-values for each SNP effect are plotted against the SNP position on each chromosome. Chromosomes are differentiated by colors. The color keys are given below the plot. The blue line indicates the threshold of the –log_10_P-values for genome-wide significance after correcting for multiple testing.

The genome-wide association study (GWAS) for ERU-affection and ERU severity score with a general linear model (GLM) revealed each one significant −log_10_P-value after correcting for multiple testing using the Bonferroni procedure (Figure 1 and 2). The significantly ERU-associated SNP (BIEC2-536712) in the case-control analysis is located on horse chromosome (ECA) 20 at 49,349,056 bp and reached a −log_10_P-value of 6.25 in the general linear model. We determined the significance threshold at a −log_10_P-value of 5.87 which corresponds to a P-value of 0.05 after applying the Bonferroni correction for multiple testing. The –log_10_P-value corrected for multiple testing was at 1.69. For the ERU severity score, the SNP BIEC2-421990 on ECA18 at 81,856,374 bp was significantly associated in the GLM analysis with a −log_10_P-value at 6.78 and a corresponding –log_10_P-value at 2.21 after Bonferroni correction. A mixed linear model (MLM) analysis confirmed the significant associations found with the GLM analysis. The corresponding –log_10_P-values in the MLM analysis were at 4.44 for the SNP BIEC2-536712 and at 5.37 for the SNP BIEC2-421990. The SNPs BIEC2-536712 and SNP BIEC2-421990 explained 22.7 and 15.5% of the phenotypic variance of ERU-affection and of the ERU severity score, respectively. Using a random sample of German warmblood horses as an extended control group confirmed the associations of these two SNPs with ERU. The observed −log_10_P-values were plotted against the expected −log_10_P-values and the quantile-quantile plots indicated that the population stratification was eliminated through the model employed (Figure S1–S2).

The highest associated SNP BIEC2-536712 in the case-control analysis is intergenic, with *interleukin 17A* (*IL-17A*) being the closest gene, approximately 514 kb upstream of *IL-17A*. Adjacent to *IL-17A* at 49,863,357–49,866,252 bp, *IL-17F* is located at 49,908,274–49,909,879 bp. The MAF of this highly associated SNP was 0.20 (G) for all 144 German warmblood horses, 0.26 for ERU-affected and 0.13 for control horses (Table 1). The OR of the minor allele was 2.45 with 95% confidence intervals at 1.24–4.86. Using the data set with the extended controls, the MAF in controls was at 0.18, the OR was 1.69 with 95% confidence intervals at 1.05–2.74.

**Table 1:** Summary of results for the genome-wide association study using a general linear model analysis for equine recurrent uveitis (ERU) in German warmblood horses.

ECA	Position	SNP-ID	Minor	MAF	MAF_a_	MAF_u_	OR	CI-L	CI-U	−log_10_P
			allele							
18	81,856,374	BIEC2-421990	A	0.18	0.33	0.12	3.65	1.61	8.26	6.78
20	49,349,056	BIEC2-536712	G	0.20	0.26	0.13	2.45	1.24	4.86	6.25

The SNP-ID, the position on horse chromosome (ECA) in base paires (bp), minor allele, minor allele frequency (MAF) for all, affected (MAF_a_) and unaffected (MAF_u_) horses and −log_10_P-values (−log_10_P) from the general linear model analysis as well as odds ratios (OR) with 95% confidence intervals (CI) from a case-control for ERU-affected (BIEC2-536712) and severely ERU-affected horses (BIEC2-421990) are given.

The SNP BIEC2-421990 with the highest association for the ERU score was located within intron 5 of *pleckstrin homology domain containing, family M, member 3* (*PLEKHM3*) at 81,810,987–81,957,457 on ECA18. Downstream to *PLEKHM3* at 82 Mb, the crystalline gene *CRYGB* is annotated on the equine genome reference assembly EquCab2.0 (http://www.ensembl.org/Equus_caballus/). In human, this syntenic region harbours the crystalline gene cluster including *CRYGA to CRYGF* and a quarter gene fragment, *CRYGG*. The MAF of this highly associated SNP was 0.18 (A) for all 144 German warmblood horses, 0.12 for control horses, 0.19 for mildly ERU-affected horses (score 1), 0.22 for moderately ERU-affected horses (score 2), 0.33 for severely ERU-affected horses (score 3) and 0.12 for control horses. The OR of the minor allele for severely ERU-affected horses was 3.65 with 95% confidence intervals at 1.61–8.26. Using the data set with the extended controls, the OR for severely ERU-affected horses was 2.26 with 95% confidence intervals at 1.19–4.32.

For both significant SNPs their flanking genomic regions with genes annotated on the horse genome assembly EquCab2.0 are shown in Figures S3–S4. We analysed the haplotype structure and haplotype association of the genomic regions flanking the associated SNPs. For the haplotype association analyses, severe ERU affection was treated as a binary trait considering severely ERU-affected horses as cases and horses free from ERU as controls. On ECA18, only one haplotype block significantly (P-value = 0.016) associated with severely ERU-affected horses could be identified. This haplotype block at 81,855,169–82,077,699 bp extends over 222 kb and includes the genome-wide significantly associated SNP BIEC2-421990. On ECA20, the significantly ERU-associated SNP and the nearest downstream SNP were contained in a haplotype block of 30 kb. For this haplotype block a significant marker-trait association at a P-value = 0.029 was found. A haplotype combining the significantly ERU-associated SNP BIEC2-536712 and the SNP BIEC2-537252 at 49,824,385 bp gave the highest haplotype association with a P-value = 0.002 from all SNPs in this genomic region.

## Discussion

The aim of this work was to identify associated regions for ERU in warmblood horses. The present results indicate that the response to ERU is associated with a genomic region located very closely to the candidate genes *IL-17A* and *IL-17F*. The haplotype containing the SNP located nearest to *IL-17A* resulted in the highest haplotype association for this ECA20 region. The final marker set did not contain SNPs located within these candidate genes and all other SNPs were more distant to the candidate genes *IL-17A* and *IL-17F*. Therefore, we assume that *IL-17A* and *IL-17F* are strong candidates involved in the development of ERU in warmblood horses. Resistance to experimental autoimmune uveitis (EAU) could be increased in mice deficient for IL-17, IL-6 and IL-23 indicating critical roles for these cytokines in pathogenesis of EAU [31]. The pro-inflammatory cytokine IL-17 has been associated with cellular damage during autoimmune diseases including uveitis in man and mice [32] as well as horses [22]. The unique cytokine profile of Th17 cells that includes IL-17A, IL-17F, IL-22 and IL-6 has lead to the identification of Th17 cells as a pathogenic IL-17 secreting CD4+ helper T-cell population, predominantly present in the uvea of ERU-affected horses [22], [25]. This cell population is considered as the main contributor to autoimmune inflammations [33]. A reduced reactivity of the cytokines IL17A and IL17F has been supposed to play a role in inducing resistance to ERU [25].

In a Chinese Han population, polymorphisms of the *IL-17F* gene were associated with the Vogt-Koyanagi-Harada syndrome (VKHS) [34] and in Korean patients with Behçet’s disease (BD) [35]. An interaction among *IL-17A*, *IL-23R* and *STAT4* polymorphisms conferred susceptibility to intestinal BD in Korean patients whereby *IL-17A* haplotypes had a positive association [36]. Upregulation of IL-17 levels in peripheral blood mononuclear cells (PBMCs) of patients with BD [37] and VKHS [38]–[40] was observed in several studies in man.

The present study is a first step to unravel the role of genetic variants in the aetiopathogenesis of ERU. Further research is necessary to show the direct relationships among genetic variants influencing the expression of IL-17A and IL-17F and the development of ERU. The previously reported linkage of ERU with the equine histocompatibility system (MHC) in Appaloosas [29] and an association using serological MHC testing in German warmblood horses [28] was substantiated through the present study.

Further genetic variants may be of importance because Th1 and Th17 cells have implications for inducing autoimmune reactions in uveitis [25]. In VKHS patients, IL-23 plays a pivotal role through inducing the IL-17 producing CD4+ T cells [38]. RANTES-like protein plays a role in the recruitment of T lymphocytes into the eyes of horses with uveitis [41]. An increased expression of the chemokine *RANTES* (*regulated upon activation, normal T-cell expressed and secreted*) had been observed in the ciliary body of eyes with recurrent uveitis [41]. IL-6 plays an indirect role in the recruitment and differentiation of Th17 cells via *RANTES*, and directly via macrophage signalling. Recent studies have implicated SOCS-3 (cytokine signalling 3) and IL-10 as negative regulators for IL-17 secretion [42]. In order to detect possible associations caused by further genetic variants, a larger design of the GWAS is necessary.

The association with the SNP BIEC2-421990 near to the crystalline gene cluster on ECA18 may indicate that ERU-affected horses may show more severe signs in presence of genetic variants influencing formation of crystalline proteins. A one base pair deletion within *CRYGE* is tightly associated with the dominant murine Elo (eye lens obsolescence) phenotype characterized by malformation of the eye lens [43]. A similar mechanism might also cause more severe signs in ERU-affected horses. Alternatively, horses exhibiting more severe signs of ERU may have overlaying cataract formation and before signs of uveitis become apparent, cataract formation may not be noticed. Cataract formation in the course of ERU is not uncommon [2], [7]–[12], [19].

In summary, this the first study showing significant associations with ERU using a GWAS in warmblood horses. A possible role for IL-17A and IL-17F is suggestive due to the co-localization of the ERU-associated SNP BIEC2-536712. The association of the SNP BIEC2-421990 close to the crystalline gene cluster on ECA18 may indicate concomitant mutations in crystalline genes and thus, lead to more severe signs in ERU-affected horses.

## Materials and Methods

### Ethics Statement

All animal work has been conducted according to the national and international guidelines for animal welfare. The Lower Saxony state veterinary office at the Niedersächsisches Landesamt für Verbraucherschutz und Lebensmittelsicherheit, Oldenburg, Germany, was the responsible Institutional Animal Care and Use Committee (IACUC) for this specific study. The EDTA-blood sampling for the present study had been approved by the IACUC of Lower Saxony, the state veterinary office Niedersächsisches Landesamt für Verbraucherschutz und Lebensmittelsicherheit, Oldenburg, Germany (registration number 33.9-42502-05-10A070).

### Animals

Clinical data and EDTA-blood samples of ERU-affected horses were collected in the Clinic for Horses of the University of Veterinary Medicine Hannover and further four veterinary clinics for horses in Northern Germany. All cases of ERU were clinically and ophthalmologically examined by veterinary experts for equine ophthalmology. We distinguished unaffected horses and ERU-affected horses with mild, moderate and severe signs. A total of 144 samples including 79 ERU-affected and 65 unaffected horses were available for the genome-wide association study. The data included 25 horses with a mild form of ERU, 30 horses with a moderate form of ERU and 24 horses with severe signs of ERU. A random sample of 286 German warmblood horses was used as an extended control group. For these horses, eye examinations were done on studs to make sure that these horses were free from ERU and did not have a veterinary report for ERU. This additional random sample of horses had been sampled on many different warmblood horse studs in Northern Germany. This additional sample of 286 horses was unrelated at the grandparent level to the detection sample of 144 horses. Age at examination was on average 15±3 years in controls and ERU-affected horses had a mean age of 10±5 years. Bay and black horses made up about 75% of the controls and about 86% of the ERU-affected horses. We collected the pedigree data for all horses to identify their ancestors up to eight generations in order to avoid stratification according to families in the horses genotyped for the GWAS.

Clinical diagnosis was based on multiple distinct inflammatory episodes in one or both eyes causing characteristic and chronic signs for ERU. All horses included in the present study as cases had at least a second episode of ocular inflammation after a period without any observable ocular inflammation. Controls had to be free from any signs of ocular diseases and any other disease. Differential diagnosis taken into account include virus keratitis, immune-mediated keratitis, ulcerative keratitis, conjunctivitis, glaucoma, stromal abscesses, uveitis due to blunt or sharp trauma or septicaemia and single episode type uveitis.

Examination of both eyes started at daylight and with a focal illumination. First, menace response and dazzle reflex were tested for a rough assessment of vision. Both the bulbar and palpebral conjunctivae, the conjunctiva overlaying the third eyelid and the palpebral surface of the third eyelid were examined. In order to make corneal lesions visible, cornea was tinted. Simultaneous digital palpation of both eyes through the upper eyelids was performed to test intraocular tension. If an elevation of intraocular pressure was suspected, the pressure was measured with a tonometer. The further examinations were performed in a dimmed environment. First, direct and indirect pupillary responses were checked. Afterwards, a direct ophthalmoscope was used in order to examine retina, optic disc and vitreous. Slit-lamp examination was performed to examine cornea, anterior chamber and lens. If the ocular fundus was not visible because of corneal oedema, large-area synechiae, cataract or opacities in the vitreous, an ultrasound scan of the eye was performed.

We distinguished three scores (1–3) for ERU-affected horses. First, scoring was done separately for each eye for iris, lens, vitreus, fundus and all the other compartments of the eye including the anterior chamber, bulbus and cornea. For each compartment and eye, a score was obtained. The highest score obtained for any of the compartments of one or both eyes was used to classify the severity of ERU for a horse.

Score 1 describes a mild form of ERU. Signs may include small focal synechia, focal capsular cataract, focal iris residue, focal chorioretinopathy, slightly depigmentation of the iris, slightly liquefaction of the vitreous, slightly visible strands of climbed cells or inflammatory products and slightly decreases of the bulb or of the anterior chamber. Score 2 characterizes moderate signs of ERU. For this category, focal large-area or multifocal synechiae; multifocal, local vesicular or local reticular capsular or subcapsular cataract or local immature cortical or nuclear cataract, moderately depigmentation of the iris, moderately liquefaction of the vitreous, moderately visible strands of climbed cells or inflammatory products, multifocal iris residues, moderately increase or slightly enlargement of the bulb or of the anterior chamber, multiple focal or peripapillary chorioretinopathies or a slightly increased intraocular pressure with local corneal opacity may be present. Severe forms of ERU were rated in case of iris atrophy, circular synechiae or seclusio pupillae, high-grade depigmentation of the iris, diffuse immature, mature or hypermature cataract, luxation as well as subluxation of the lens, high-grade liquefaction of the vitreous, high-grade visible strands of climbed cells or inflammatory products and yellow haze, large chorioretinopathies, wrinkled retina, ablation retinae, high-grade enlargement of the bulb or of the anterior chamber with high-grade increased intraocular pressure, Haab`s striae of the cornea or phtisis bulbi.

### Genotyping

For genotyping we isolated genomic DNA from EDTA blood samples using standard methods with RBC (Red Blood Cell) lysis buffer and SE (sodium EDTA) buffer. The DNA concentration of the samples was adjusted to 50 ng/µl. DNA concentration was determined using the Nanodrop ND-1000 (Peqlab Biotechnology, Erlangen, Germany).

Genotyping was performed using the Illumina equine SNP50 beadchip including 54,602 SNPs (single nucleotide polymorphisms) using standard procedures as recommended by the manufacturer. Data was analysed using the genotyping module version 3.2.32 of the BeadStudio program (Illumina). With the help of BeadStudio software a cluster file was generated.

### Data Analysis

A total of 37,040 informative SNPs with a minor allele frequency (MAF) of >0.05, a call rate of >90% and an average MAF of 0.25 were left for the analysis of the German warmblood horses. Data quality control was done using PLINK version 1.07 (http://pngu.mgh.harvard.edu/purcell/plink/) [44] and SAS/Genetics version 9.3 (Statistical Analysis System, Cary, NC, USA, 2013). Genotyping rate per animal was >0.98. The threshold for Hardy-Weinberg equilibrium was set to a P-value of 10^−7^.

For the GWAS, a case-control association analysis was performed for German warmblood horses. In addition, a score on a scale from 0 (unaffected) to 3 (severely affected by ERU) was used as trait variable. General and mixed linear models (MLM) were employed for the genome-wide association analysis in order to control for a complex population structure, sex and coat colour of the horses. The analyses were performed using TASSEL (Trait Analysis by Association, Evolution and Linkage), version 3.0.146, a software appropriate for association mapping of complex traits in diverse samples [45]. A marker based identity-by-state (IBS) kinship matrix among all horses (K-matrix) was employed for parameterization of a random polygenic effect. The Q-matrix to explain population structure was estimated using equidistantly distributed SNPs at a pairwise linkage disequilibrium (r^2^) <0.3. This pruned set of SNPs was generated using a window size of 50 SNPs, a shift by five SNPs at each step and an r^2^<0.3. The number of SNPs contained of this subset was 14,547. The Q-matrix was determined using STRUCTURE, version 2.3.3 [46]. The final MLM model included the effects of sex as class variable and the random animal effects parameterized via the IBS kinship matrix and the respective genotype effect. We also tested several extended to show the robustness of the outcome of the GWAS. All these models yielded the same highly significant associated SNP as the final model. Thus, adding principal components for cryptic data structure or the effects of the age of examination or coat colour did not change the results of the final model. The effect of coat colour was tested as the data were not completely balanced by black, bay and chestnut horses. Phenotypic variance explained by ERU-associated SNPs was calculated using the final model of the MLM analysis. Quantile-quantile (Q–Q) plots for observed versus expected –log_10_P-values were constructed to control for population stratification and to visualize significant SNP genotype effects. Significance thresholds were determined using a Bonferroni correction and the MULTITEST procedure of SAS.

For the genomic regions containing SNPs with the highest −log_10_P-values, we estimated the size of haplotypes for ERU-affected horses using LD and haplotype block analysis by Haploview, version 4.2 [47].

## Supporting Information

Figure S1 **Q–Q plot of observed versus expected** −**log_10_P-values from a genome- wide association analysis for equine recurrent uveitis (ERU) in German warmblood horses.** The quantile-quantile plot shows the expected distribution (solid line) and the observed −log_10_P-values plotted against the expected −log_10_P-values (black dots). The highest −log_10_P-value achieved significance after correcting for multiple tests using the Bonferroni procedure.
(DOC)

Figure S2 **Q–Q plot of observed versus expected** −**log_10_P-values from a genome- wide association analysis using a score for the severity of equine recurrent uveitis (ERU) in German warmblood horses.** The quantile-quantile plot shows the expected distribution (solid line) and the observed −log_10_P-values plotted against the expected −log_10_P-values (black dots). The highest −log_10_P-value achieved significance after correcting for multiple tests using the Bonferroni procedure.
(DOC)

Figure S3 **Haplotype structure for horse chromosome 18 at 79,996,485–82,495,575 bp and corresponding genes annotated on the horse genome reference assembly EquCab2.0 (** http://www.ensembl.org/Equus_caballus/ **).** The haplotype block 3 containing the SNP BIEC2-421990 associated with severe ERU-affected horses is significantly (P-value = 0.016) associated with severe ERU. The figure displays Hedrige’s multialleic D, which represent the degree of linkage disequlibrium between each two SNPs. Red fields display LOD≥2 (D’ = 1), shades of red show the same LOD with D’<1. White and blue fields display LOD<2 with D’<1 and D’ = 1.
(DOC)

Figure S4 **Haplotype structure for horse chromosome 20 at 47,360,615–51,173,997 bp and corresponding genes annotated on the horse genome reference assembly EquCab2.0 (** http://www.ensembl.org/Equus_caballus/ **).** The haplotype block 7 containing the ERU-associated SNP BIEC2-536712 is significantly (P-value = 0.029) associated with ERU. The haplotype with the SNPs BIEC2-536712 and BIEC2-537252 showed the highest haplotype association with a P-value = 0.002. The SNP BIEC2-537252 is located 38,972 bp upstream to *IL-17A*. The figure displays Hedrige’s multialleic D, which represent the degree of linkage disequlibrium between each two SNPs. Red fields display LOD≥2 (D’ = 1), shades of red show the same LOD with D’<1. White and blue fields display LOD<2 with D’<1 and D’ = 1.
(DOC)
