# Supplementary material for: A Genome-Wide Association Study Identifies Risk Loci to Equine Recurrent Uveitis in German Warmblood Horses
Source: PLoS One. 2013 Aug 14;8(8):e71619. doi: 10.1371/journal.pone.0071619 (PMC3743750; doi:10.1371/journal.pone.0071619)
Supplement: Figure S2 — Q–Q plot of observed versus expected −log10P-values from a genome- wide association analysis using a score for the severity of equine recurrent uveitis (ERU) in German warmblood horses. The quantile-quantile plot shows the expected distribution (solid line) and the observed −log10P-values plotted against the expected −log10P-values (black dots). The highest −log10P-value achieved significance after correcting for multiple tests using the Bonferroni procedure. (DOC) [file pone.0071619.s002.doc]

**
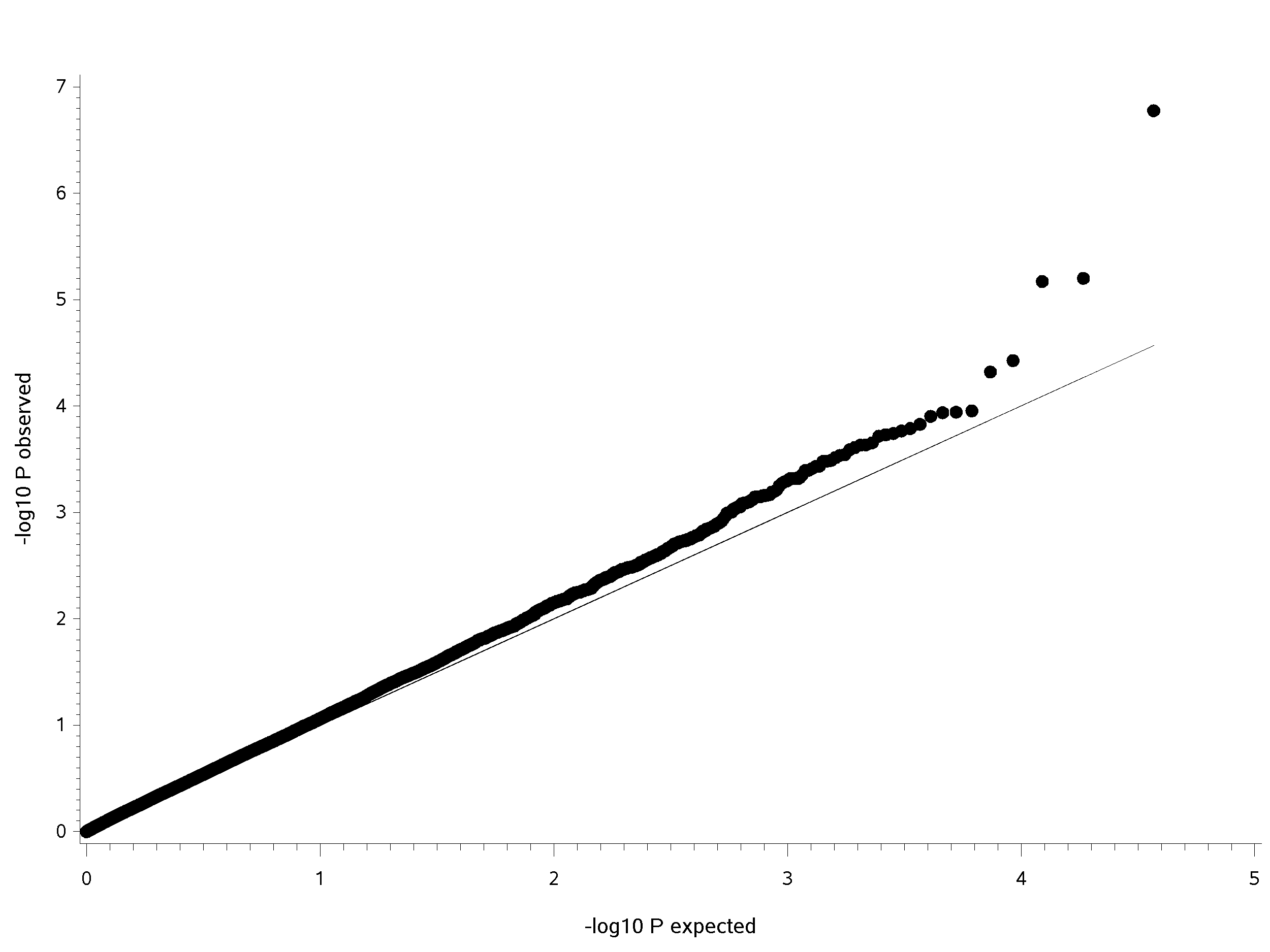
**

**Figure S2. Q-Q plot of observed versus expected -log_10_P-values from a genome- wide association analysis using a score for the severity of equine recurrent uveitis (ERU) in German warmblood horses.** The quantile-quantile plot shows the expected distribution (solid line) and the observed -log_10_P-values plotted against the expected -log_10_P-values (black dots). The highest -log_10_P-value achieved significance after correcting for multiple tests using the Bonferroni procedure.
