# Supplementary material for: A Genome-Wide Association Study Identifies Risk Loci to Equine Recurrent Uveitis in German Warmblood Horses
Source: PLoS One. 2013 Aug 14;8(8):e71619. doi: 10.1371/journal.pone.0071619 (PMC3743750; doi:10.1371/journal.pone.0071619)
Supplement: Figure S3 — Haplotype structure for horse chromosome 18 at 79,996,485–82,495,575 bp and corresponding genes annotated on the horse genome reference assembly EquCab2.0 ( http://www.ensembl.org/Equus_caballus/ ). The haplotype block 3 containing the SNP BIEC2-421990 associated with severe ERU-affected horses is significantly (P-value = 0.016) associated with severe ERU. The figure displays Hedrige’s multialleic D, which represent the degree of linkage disequlibrium between each two SNPs. Red fields display LOD≥2 (D’ = 1), shades of red show the same LOD with D’<1. White and blue fields display LOD<2 with D’<1 and D’ = 1. (DOC) [file pone.0071619.s003.doc]

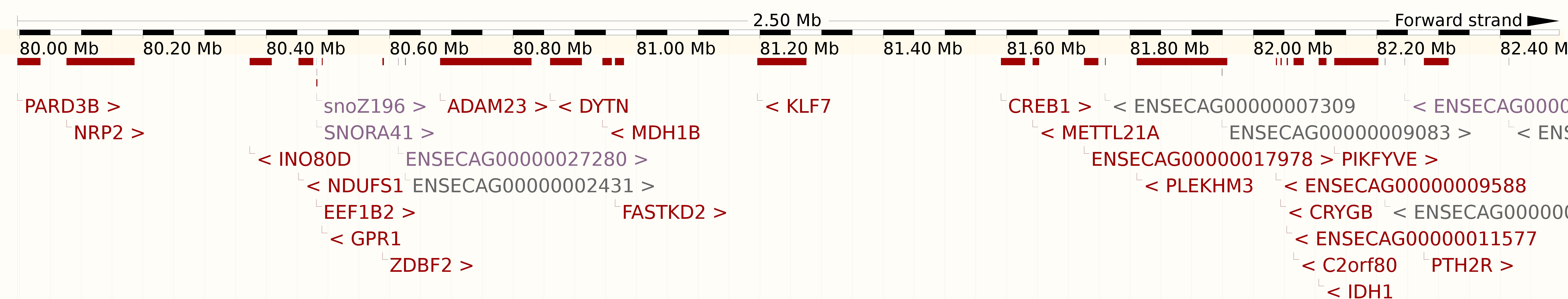


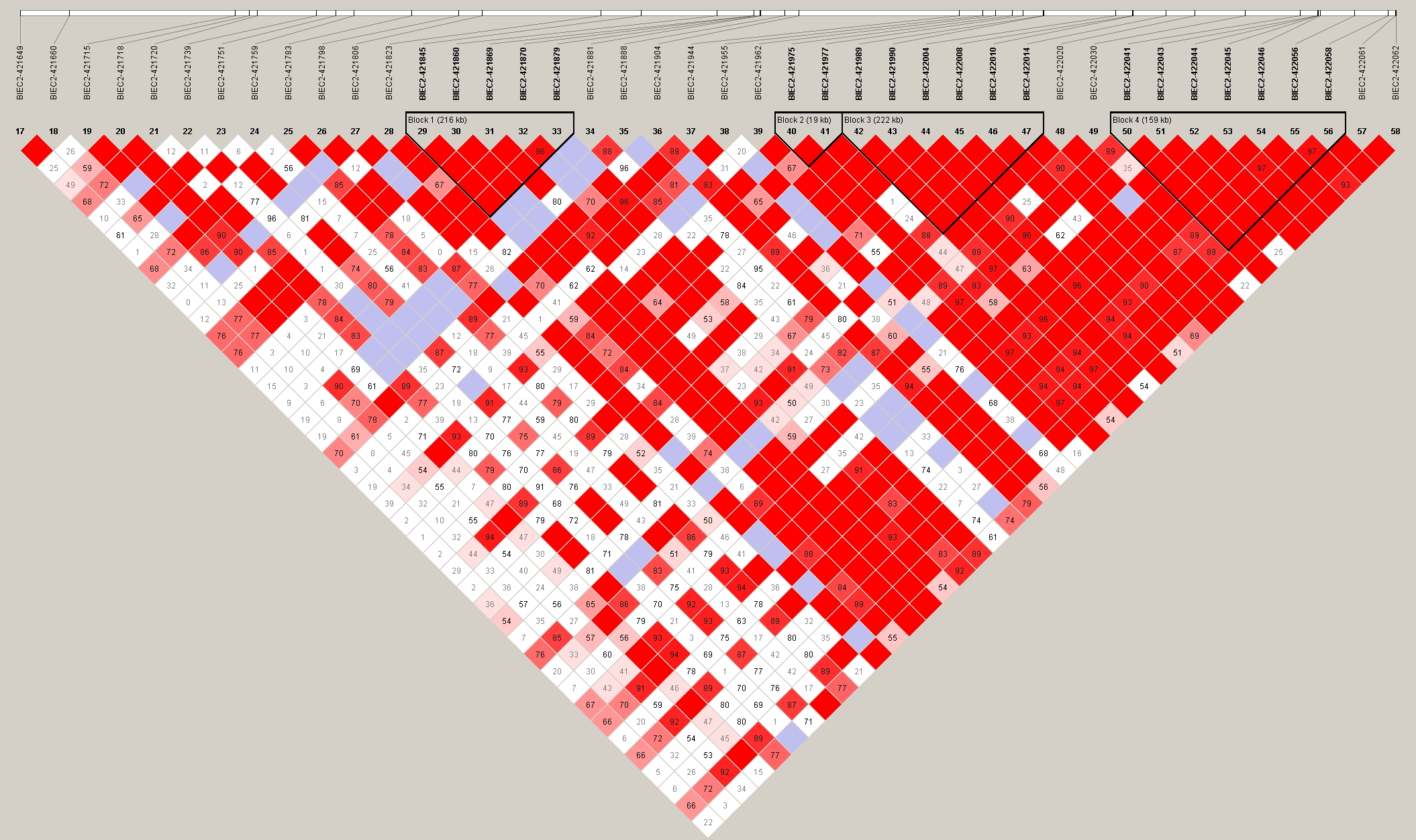


**Figure S3. Haplotype structure for horse chromosome 18 at 79,996,485–** **82,495,575 bp and corresponding genes annotated on the horse genome reference assembly EquCab2.0 (http://www.ensembl.org/Equus_caballus/).** The haplotype block 3 containing the SNP BIEC2-421990 associated with severe ERU-affected horses is significantly (P-value=0.016) associated with severe ERU. The figure displays Hedrige’s multialleic D, which represent the degree of linkage disequlibrium between each two SNPs. Red fields display LOD≥2 (D’=1), shades of red show the same LOD with D’<1. White and blue fields display LOD<2 with D’<1 and D’=1.
