# Supplementary material for: A Genome-Wide Association Study Identifies Risk Loci to Equine Recurrent Uveitis in German Warmblood Horses
Source: PLoS One. 2013 Aug 14;8(8):e71619. doi: 10.1371/journal.pone.0071619 (PMC3743750; doi:10.1371/journal.pone.0071619)
Supplement: Figure S4 — Haplotype structure for horse chromosome 20 at 47,360,615–51,173,997 bp and corresponding genes annotated on the horse genome reference assembly EquCab2.0 ( http://www.ensembl.org/Equus_caballus/ ). The haplotype block 7 containing the ERU-associated SNP BIEC2-536712 is significantly (P-value = 0.029) associated with ERU. The haplotype with the SNPs BIEC2-536712 and BIEC2-537252 showed the highest haplotype association with a P-value = 0.002. The SNP BIEC2-537252 is located 38,972 bp upstream to IL-17A. The figure displays Hedrige’s multialleic D, which represent the degree of linkage disequlibrium between each two SNPs. Red fields display LOD≥2 (D’ = 1), shades of red show the same LOD with D’<1. White and blue fields display LOD<2 with D’<1 and D’ = 1. (DOC) [file pone.0071619.s004.doc]

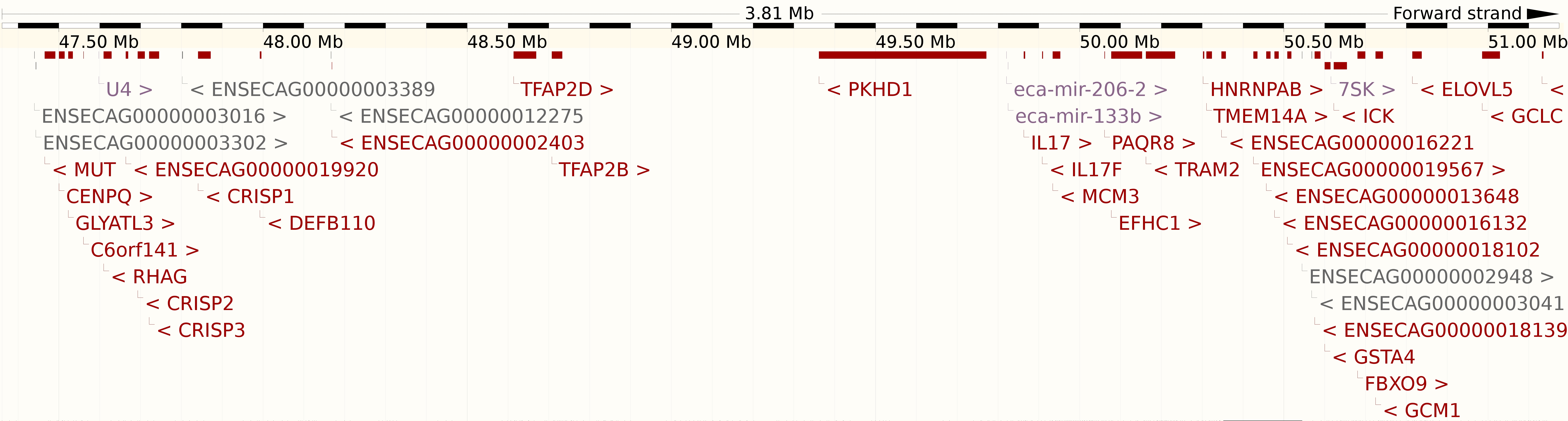


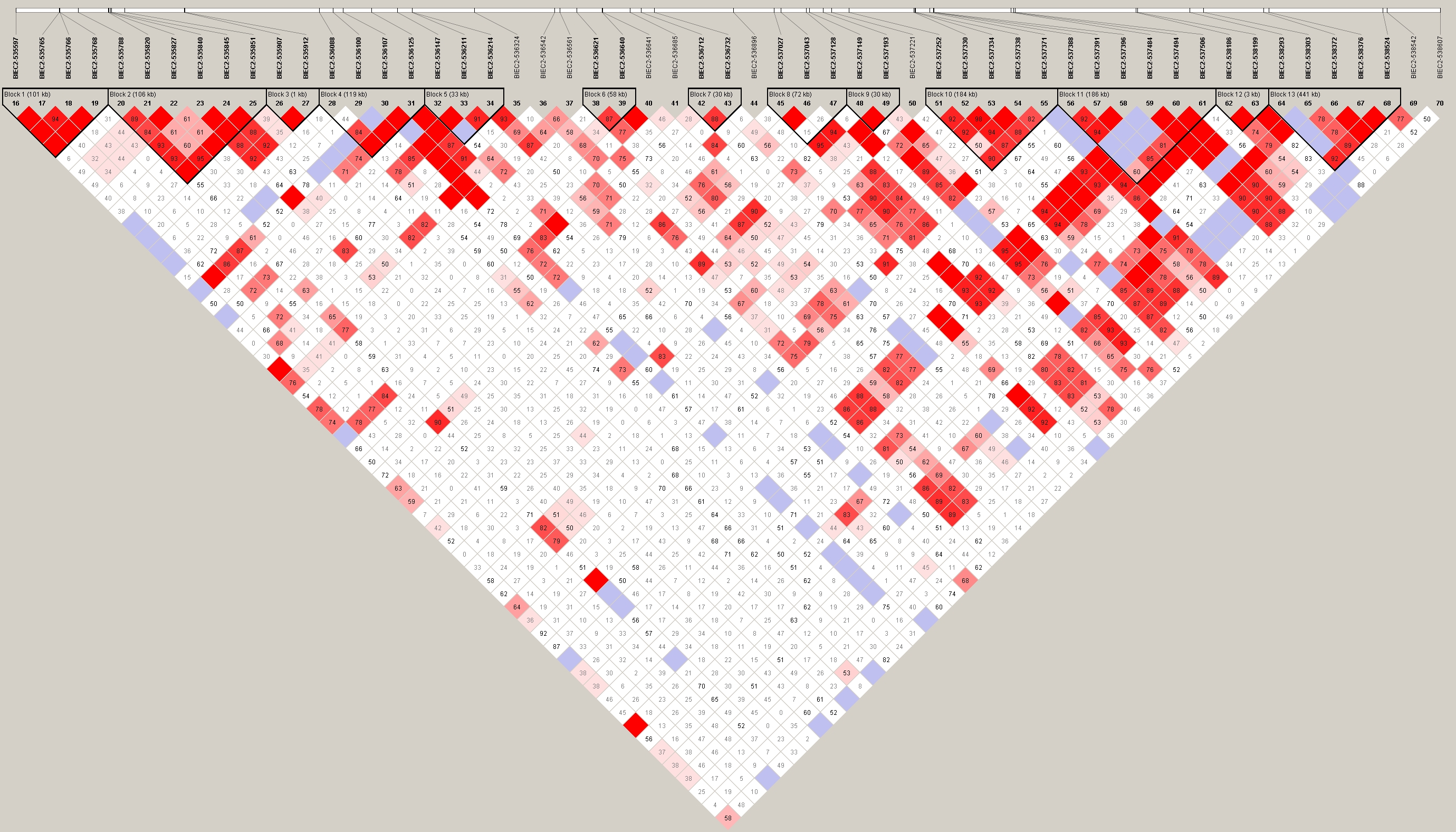


**Figure S4. Haplotype structure for horse chromosome 20 at 47,360,615–51,173,997 bp and corresponding genes annotated on the horse genome reference assembly EquCab2.0 (http://www.ensembl.org/Equus_caballus/).** The haplotype block 7 containing the ERU-associated SNP BIEC2-536712 is significantly (P-value=0.029) associated with ERU. The haplotype with the SNPs BIEC2-536712 and BIEC2-537252 showed the highest haplotype association with a P-value=0.002. The SNP BIEC2-537252 is located 38,972 bp upstream to *IL-17A*. The figure displays Hedrige’s multialleic D, which represent the degree of linkage disequlibrium between each two SNPs. Red fields display LOD≥2 (D’=1), shades of red show the same LOD with D’<1. White and blue fields display LOD<2 with D’<1 and D’=1.
